# Influencing factors of corticomuscular coherence in stroke patients

**DOI:** 10.3389/fnhum.2024.1354332

**Published:** 2024-03-18

**Authors:** Zhixian Gao, Shiyang Lv, Xiangying Ran, Yuxi Wang, Mengsheng Xia, Junming Wang, Mengyue Qiu, Yinping Wei, Zhenpeng Shao, Zongya Zhao, Yehong Zhang, Xuezhi Zhou, Yi Yu

**Affiliations:** ^1^School of Medical Engineering, Xinxiang Medical University, Xinxiang, China; ^2^Engineering Technology Research Center of Neurosense and Control of Henan Province, Xinxiang, China; ^3^Henan International Joint Laboratory of Neural Information Analysis and Drug Intelligent Design, Xinxiang, China

**Keywords:** stroke, corticomuscular coherence, EEG, sEMG, influencing factors

## Abstract

Stroke, also known as cerebrovascular accident, is an acute cerebrovascular disease with a high incidence, disability rate, and mortality. It can disrupt the interaction between the cerebral cortex and external muscles. Corticomuscular coherence (CMC) is a common and useful method for studying how the cerebral cortex controls muscle activity. CMC can expose functional connections between the cortex and muscle, reflecting the information flow in the motor system. Afferent feedback related to CMC can reveal these functional connections. This paper aims to investigate the factors influencing CMC in stroke patients and provide a comprehensive summary and analysis of the current research in this area. This paper begins by discussing the impact of stroke and the significance of CMC in stroke patients. It then proceeds to elaborate on the mechanism of CMC and its defining formula. Next, the impacts of various factors on CMC in stroke patients were discussed individually. Lastly, this paper addresses current challenges and future prospects for CMC.

## 1 Introduction

**Figure 1:**
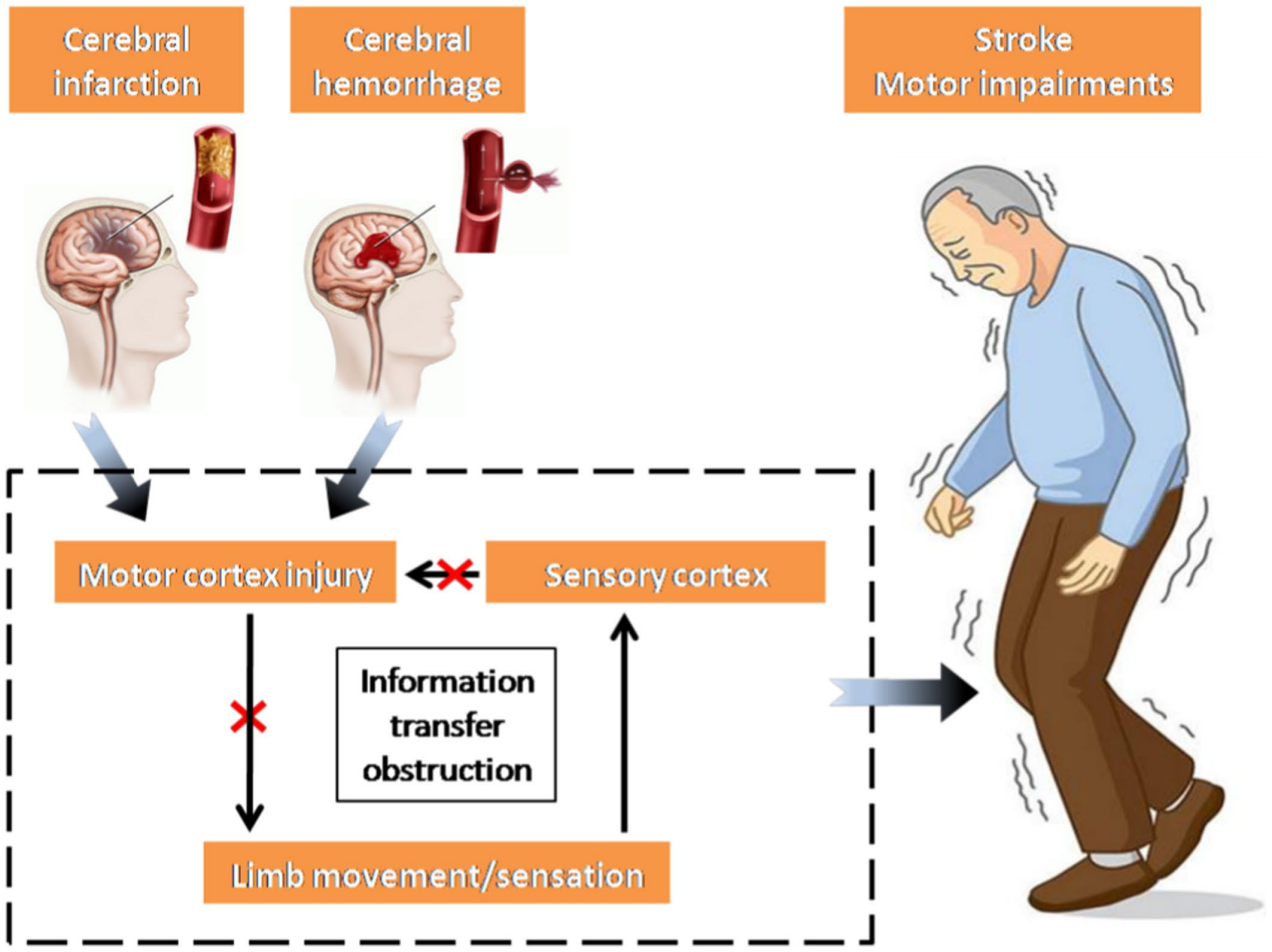
Mechanism of movement disorders in stroke patients.

Stroke, also known as a cerebrovascular accident, refers to a group of acute diseases where brain tissues are deprived of normal blood supply due to ruptured or blocked blood vessels, leading to subsequent brain tissue damage (Fang et al., 2022). Following a stroke, inadequate blood supply to the brain hinders the delivery of sufficient nutrients to the brain tissue, leading to neurological impairment (Dabrowska et al., 2019). Once the damage affects the motor cortex, the transmission of nerve impulses carrying motor commands is disrupted, leading to impaired transmission from the brain to the muscles, ultimately causing motor dysfunction in the limbs. Simultaneously, the motor information from the limbs, conveyed through the sensory cortex, cannot be promptly regulated by the impaired motor cortex, thus intensifying the motor impairments observed in stroke patients (Sun et al., 2014; Chen et al., 2018a; Li et al., 2022; Figure 1).

In essence, the primary factor behind movement disorders in stroke patients is the irregular transmission of nerve oscillations. This disruption impacts the connectivity of the corticospinal pathway between the cerebral cortex and muscle, consequently diminishing the neural control exerted by the brain on the muscle (Choi, 2016). Nonetheless, the clinical assessment of motor impairment and functional recovery in stroke patients predominantly relies on diverse scales and the expertise of physicians, resulting in a less efficient and more subjective approach (Jalloul, 2018). An index is required to measure the interaction between the cerebral cortex and controlled muscle activity, facilitating an objective assessment of motor function recovery post-stroke. Consequently, corticomuscular coherence (CMC) has been introduced as one of the effective approaches to examine the neural oscillations of the cortex and muscles. Its purpose is to objectively assess the functional connectivity of cerebral muscles in stroke patients. The discovery of CMC initially occurred in a case involving treatment-resistant epilepsy, where electroencephalography (EEG) and surface electromyography (sEMG) were employed (McLachlan and Leung, 1991). Large individual differences exist in CMC itself (Ushiyama et al., 2011). A significant CMC may indicate utilization of the corticospinal pathway during voluntary contraction, whereas a non-significant CMC does not conclusively suggest non-utilization of the pathway. Instead, it may imply variation in synchronous oscillation strategies between the cortex and muscles of participants, thereby affirming the utility of CMC in studying abnormal neural oscillation transmission in the corticospinal pathway in stroke patients.

Currently, many scholars apply CMC in studying stroke patients to elucidate the pathological mechanisms underlying motor dysfunction. Techniques such as EEG and magnetoencephalography (MEG) (Conway et al., 1995) or functional magnetic resonance imaging (fMRI) (Ward et al., 2003) are employed. However, fMRI suffers from low temporal resolution, limiting its ability to capture neural activity and muscle responses during rapid movements, potentially leading to loss of detail in the study of brain-muscle interaction (Farr et al., 2016; Rojas et al., 2018). Furthermore, motor execution tasks commonly used in studying CMC in stroke patients can introduce significant motor artifacts that heavily impact fMRI results (Dash et al., 2019; Kuhara et al., 2023). Conversely, MEG, while expensive and less clinically accessible, offers higher temporal resolution. Thus, the prevalent method for analyzing CMC in stroke patients involves synchronous EEG and surface electromyography (sEMG) to investigate brain-myoelectric coupling, facilitating the study of abnormal neural oscillations and cortical muscle control responses post-stroke (Ito et al., 2021). This approach enhances our understanding of stroke pathology and provides a scientific basis for stroke rehabilitation treatment.

The primary sections of this paper include: the CMC definition formula and improvement, which presents an extensive definition of CMC and a method for its enhancement; an in-depth analysis of the factors impacting CMC in stroke patients, encompassing pathological state, rehabilitation training, and experimental design; and the Existing Problems and Prospects section, which outlines the current issues in CMC research and the envisioned future direction.

## 2 The CMC definition formula and improvement

Coherence serves as an extension of the Pearson correlation coefficient in the frequency domain. It is defined as the ratio of the auto-spectrum to the cross-spectrum and provides a normalized correlation measure, denoted as a real number between 0 and 1. A value of 1 signifies complete linear correlation between two signals, while a value of 0 indicates no linear correlation whatsoever (Mima and Hallett, 1999). Assuming the existence of two signals, x and y , the coherence value (CMC) at a signal frequency f is calculated as follows:


(1) $${∁}_{xy}\left(f\right)=\frac{{\left|P_{xy}\left(f\right)\right|}^{2}}{\left|P_{xx}\left(f\right)\right||P_{yy}\left(f\right)|}$$

(2) $$P_{xy}\left(f\right)=\overset{N-1}{\underset{m=0}{\sum }}R_{xy}\left(m\right)e^{-jfm}$$

In Equation (1), $C_{xy}\left(f\right)$ represents the coherence value of the two signals at frequency f , while $P_{xy}\left(f\right)$ denotes the cross-spectrum at frequency f for signals x and y , and $P_{xx}\left(f\right)$ or $P_{yy}\left(f\right)$ represents the auto-spectrum for signal x or signal y at frequency f . These power spectra are calculated using Equation (2). Here, $R_{xy}\left(m\right)$ denotes the cross-spectrum sequence signals of the time series for x and y , with m indicating the cross-spectrum sequence signal of the first m coordinate of the sampling point. When x represents the EEG signal and y represents the sEMG signal, CMC can be used to assess their linear correlation in the frequency domain, providing a quantitative evaluation of the correlation between different neural regions.

EEG signals consist of complex frequency components and are influenced by numerous factors. Existing coherence analysis methods rely on estimating the spectral density function through the Fourier transform (Carter and Knapp, 1973), leading to inter-spectral interference that impact the accuracy of coherence spectral estimation for the β and γ primary functional frequency bands. Consequently, Ma et al. (2014) proposed a coherence analysis method based on wavelet decomposition for EEG and sEMG. This approach involves decomposing EEG signals into distinct frequency subbands and selecting the functional frequency bands (β and γ bands) associated with muscle motor control in both EEG and sEMG signals. This method more comprehensively elucidates the distinctions in coherence across various functional frequency bands of EEG and sEMG. Synchronized coupling of EEG and sEMG signals is associated with a time delay, which, if ignored, might diminish the coherence level. Consequently, Xu et al. (2016) introduced an analytical method called corticomuscular coherence with time lag (CMCTL), which aligns more closely with physiological observations. Drawing on Horak’s motor control theory (Mazzoni et al., 2012), the bidirectional nature of the control response between the human cerebral cortex and muscles is evident, posing a challenge for CMC in determining the direction of coupling. Gao et al. (2017) presented a multifrequency band analysis method for bidirectional coupling between cerebral EMG based on coherence. This approach involved selecting EEG signals from motor area in the cerebral cortex and limb sEMG signals, as well as limb sEMG signals and EEG signals from the somatosensory area, for multifrequency band bidirectional coupling coherence analysis. The outcomes were contrasted with those of the newly introduced causality-based coupling analysis. The study revealed that the bidirectional coupling, associated with an increase in grip force, shifted to the high-frequency band, highlighting the enhanced clarity of the coherent coupling analysis method. This approach effectively illustrated the pattern of change in coupling strength across frequency bands in relation to grip force, thereby addressing the limitation of CMC in determining coupling direction. Furthermore, considering the fundamental role of motor control, the interaction between the sensorimotor cortex and peripheral nerve tissue has been demonstrated to be notably nonlinear (Darvas et al., 2009). However, CMC is limited to computing the linear correlation of signals solely within the frequency domain. Consequently, Yang et al. (2015, 2016b) introduced an n:m coherence analysis method, serving as a comprehensive coherence measure designed to assess cross-frequency coupling between two distinct frequency components. This method unveiled, for the first time, both the linear and nonlinear corticomuscular coupling between the primary sensorimotor areas, their corresponding regions, and the peripheral muscles. Additionally, the study indicated that corticospinal tracts predominantly facilitate linear corticomuscular coupling, while non-linear coupling might be associated with sensory feedback pathways. The introduction of these enhanced methods and findings offers a more precise avenue for comprehending the communication dynamics between the cerebral cortex and muscles, consequently providing a fresh perspective on the mechanisms underlying cortical muscle motor control in stroke patients.

## 3 Influencing factors

**Figure 2:**
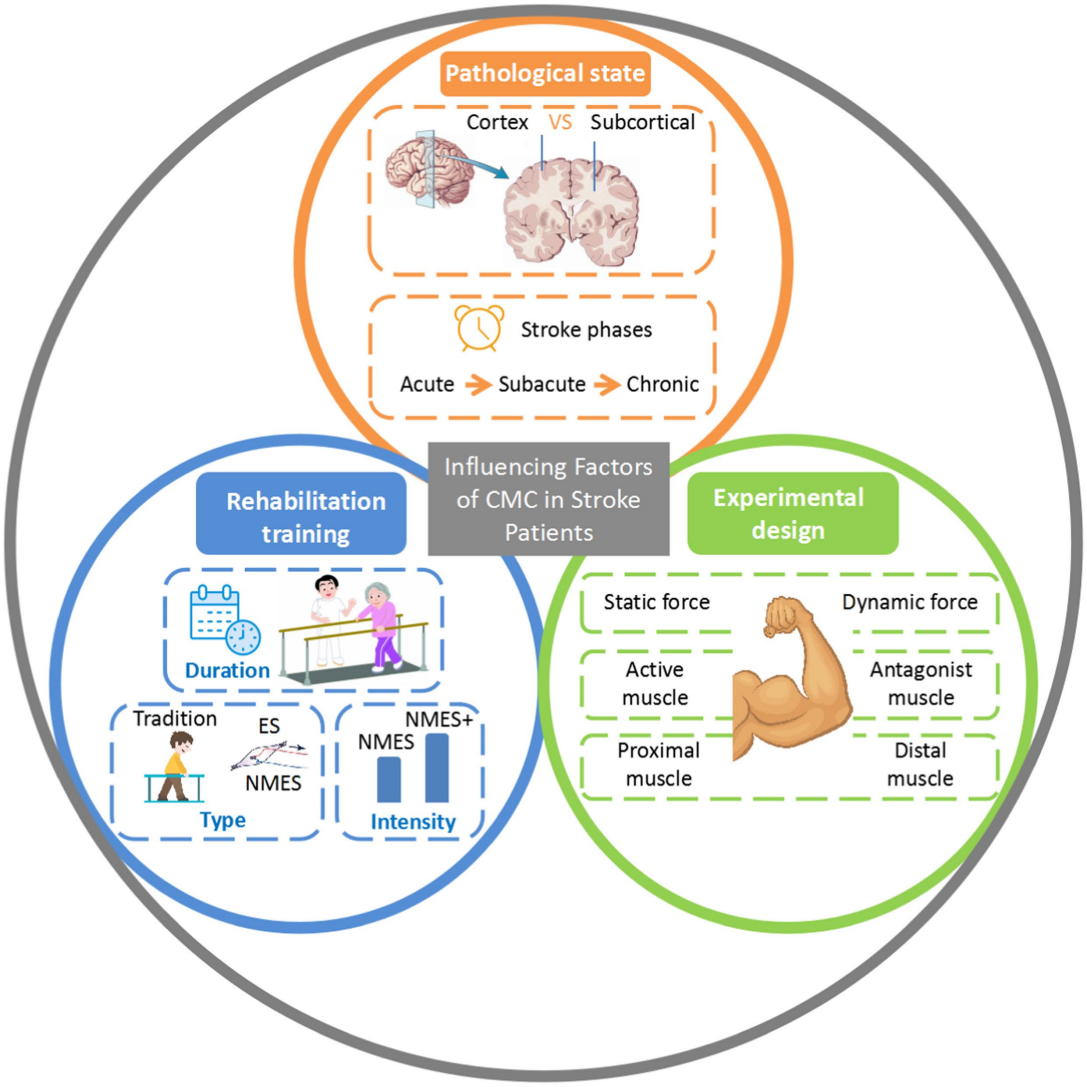
Influencing factors of CMC in stroke patients. VS, versus; CMC, corticomuscular coherence; ES, electrical stimulation; NMES, neuromuscular electrical stimulation.

Summarizing the existing research on CMC in stroke patients, this paper identifies various factors influencing CMC in this population, including pathological state, rehabilitation training, and experimental design (Figure 2). At the same time, the main references in this paper are listed in Table 1.

**Table 1:** Summary of main references on influencing factors of CMC in stroke.

Study	Country	Influencing factors of CMC in stroke patients								Main results
		Pathological state			Rehabilitation training			Experimental design		
		Healthy vs. Stroke	Stroke location	Stroke phases	Training duration	Training type	Training intensity	Motor tasks	Target muscles	
Chen et al. (2018a)	China	√								Healthy controls exhibited higher coupling
Ma et al. (2014)	China				√					The rehabilitation training led to varying degrees of improvement in motor function on the affected side, accompanied by a tendency of γ band CMC on the affected side to increase with the recovery of motor function.
Fang et al. (2009)	USA	√						√		Stroke patients had significantly lower CMC. In stroke patients engaging in an upper limb extension dynamic force task, the γ band CMC of the TB,AD, and BB muscles was notably lower compared to healthy controls.
Rossiter et al. (2012)	UK	√	√							The gamma CMC value was significantly lower in patients. Stroke patients with cortical infarcts are more likely to exhibit peak CMC on the healthy side of the brain.
Von Carlowitz-Ghori et al. (2014)	Germany	√		√						At acute stage, peak frequencies of CMC of patients were significantly smaller. During the acute phase, the peak CMC was located on the healthy side of the brain, while in the chronic phase, there was no notable distinction in CMC amplitude between the affected and healthy brain sides.
Xu et al. (2023)	China	√								The CMC values were notably lower in stroke patients.
Park et al. (2016)	Korea		√							The variations in activation levels and electroencephalographic power among the subgroups with distinct lesion sites.
Krauth et al. (2019)	Germany		√	√						In patients with pure subcortical infarction, the peak CMC appeared on either the healthy or the affected side of the brain. In patients with motor cortex infarction, the peak CMC emerged on the healthy side of the brain. CMC amplitude was lower in the acute phase and gradually surpassed levels observed in healthy individuals during the chronic phase
Zheng et al. (2018)	China			√	√					The CMC between activities from the paretic limb muscles and the contralateral motor cortex for the second time of experiment increased significantly compared with that for the first time. The significant improvements in CMC after the rehabilitation period (for four weeks) compared to before
Geertsen et al. (2013)	Denmark				√					Following 27 min of cocontraction training, the nondancers improved their performance significantly and showed a significant increase in 15- to 35-Hz coherence
Larsen et al. (2016)	Denmark				√					Following motor practice performance improved significantly and a significant increase in EEG–EMG_APB_ and EMG_APB_-EMG_FDI_ coherence in the β band (15–30 Hz) was observed.
Perez et al. (2006)	Denmark				√					A significant increase in EEG–EMG coherence around 15–35 Hz was observed following the visuo-motor skill session in nine subjects.
Belardinelli et al. (2017)	Germany				√					The upper extremity-FMA of the patients improved significantly. All patients showed significantly increased CMC in the β frequency-band.
Pan et al. (2018)	China					√				The CMC in strokes (ES group) at fourth weeks was significantly higher, and hand function was improved (ES group).
Lai et al. (2016)	China					√				After ES, EEG–EMG coherence in γ band increased significantly for 48.6% in stroke survivors, respectively.
Bao et al. (2021)	China					√	√			The combined rehabilitation approach integrating NMES and traditional methods facilitated CMC of the ipsilesional brain and paretic lower limbs. The stronger NMES enhances CMC in both the ascending and descending pathways.
Bao et al. (2019)	China					√	√			Single-session real-time sensory-level NMES during pedaling tasks could only modulate corticomuscular coupling in the ascending pathways for chronic stroke survivors.
Kwakkel et al. (2004)	Netherlands						√			Augmenting rehabilitation intensity led to enhancements in motor function within the initial 6 months following stroke.
Chen et al. (2018b)	China							√		When stroke patients performed dynamic force and static force tasks, significant CMC appeared in γ band and β band, respectively.
Guo et al. (2020)	China	√						√	√	The CMC of ED in stroke patients was significantly lower than that of healthy controls when performing finger extension tasks (20%iMVC), while there was no difference between the two groups when performing finger flexion tasks. In stroke patients who completed finger stretches with 20 to 40% iMVC, the CMC peak in FD was significantly lower than that in healthy controls, while the CMC peak in ED was not different in healthy controls.
Bao et al. (2018)	China							√		When stroke patients dynamically modify wrist extension, the γ band CMC on the affected side was notably lower compared to the healthy side.
Mima et al. (2001)	USA								√	The peak CMC for OP and ECR on the affected side was significantly lower than that on the healthy side, while the peak CMC for BB did not exhibit a significant difference between the affected and healthy sides.

CMC, corticomuscular coherence; TBt, ricepsbrachii; AD, anterior deltoid; BB, biceps brachii; EEG, electroencephalogram; EMG, electromyography; APB, abductor pollicis brevis; FDI, first dorsal interosseous; FMA, fugl-meyerassessment; ES, electrical stimulation; NMES, neuromuscular electrical stimulation; iMVC, isometric maximum voluntary contraction; FD, flexor digitorum; ED, extensor digitorum; OP, opponens pollicis; ECR, extensor carpus radialis.

### 3.1 Pathological state

Stroke refers to a cerebral injury resulting from a significant decrease in cerebral blood flow caused by either arterial bleeding (hemorrhagic stroke) or blockage (ischemic stroke). The motor control system operates as a closed-loop mechanism. Brain damage following a stroke leads to modified neural oscillations, compromised upload/download pathways, and impaired muscle function. Consequently, stroke patients manifest irregularities in various facets of CMC. Given that the self-regulation and interaction of motor control systems occur at various spatial and temporal scales, structural brain damage post-stroke could potentially disturb the coordination, feedback, and information exchange between efferent control and afferent feedback (Chen et al., 2018a). The majority of studies indicate that CMC is comparatively weaker in stroke patients than in their healthy counterparts (Fang et al., 2009; Rossiter et al., 2012; von Carlowitz-Ghori et al., 2014; Chen et al., 2018a). However, it is worth noting that the existing research on CMC in stroke patients has predominantly concentrated on upper limb functions, with limited exploration of the functional connectivity between the brain and lower limbs in this population. Xu et al. (2023) conducted a comparative analysis of the tibialis anterior (TA), lateral gastrocnemius (LG), and medial gastrocnemius (MG) muscles during unilateral static ankle dorsiflexion in both stroke patients and healthy controls. The CMC values were notably lower in stroke patients compared to the values observed in healthy controls. Brain topography revealed substantial coherence in the cortical centers among the healthy control group, whereas such coherence was not observed in the stroke patient group. However, it should be noted that the situation was not universally consistent, despite the aforementioned findings demonstrating weaker CMC in stroke patients in comparison to healthy controls.

Stroke location within the brain varies and is primarily classified as either cortical infarcts or subcortical infarcts based on the site of cerebrovascular lesions. Disparities in lesion location give rise to specific motor impairments among stroke patients (Donnan et al., 2008). For instance, damage to the central gyrus (motor cortex region) results in motor deficits or limb paralysis (Shelton and Reding, 2001). Impairment to the internal capsule (subcortical region) hinders both motor and sensory functions (Abela et al., 2012). Park et al. (2016) classified chronic stroke patients into three subgroups: “SM1+” with supratentorial lesions (including primary motor areas), “SM1-” with supratentorial lesions (excluding primary motor areas), and those with infratentorial lesions. They discovered variations in activation levels and electroencephalographic power among the subgroups with distinct lesion sites. These findings align with results from fMRI-related investigations, indicating that brain activation patterns differ depending on the lesion location (Luft et al., 2004, 2005; Alexander et al., 2009). A close association exists between CMC and brain activation, representing the synchronization and coordination between cortical activation and body muscle movement. CMC serves as a crucial indicator of brain-controlled movement. Consequently, alterations in the lesion site might impact CMC.A study investigating alterations in the peak CMC location in brain topography after stroke involved 25 stroke patients (Rossiter et al., 2012), encompassing both cortical and subcortical infarcts. Patients with CMC peaks on the affected side of the brain exhibited five cortical infarcts and four subcortical infarcts. Patients with CMC peaks on the healthy side of the brain had six cortical infarcts and two subcortical infarcts. Two patients experiencing motor cortex damage exhibited peak CMC on the healthy side of the brain. Proportionally (cortical infarcts: subcortical infarcts), it seems that stroke patients with cortical infarcts are more likely to exhibit peak CMC on the healthy side of the brain. Specifically, patients with motor cortex damage displayed all their CMC peaks on the healthy side of the brain. This aligns with Krauth et al.’s findings (Krauth et al., 2019), which involved four stroke patients, one with combined motor cortex and subcortical infarcts, and the remaining three with subcortical infarcts. The results indicated that in patients with pure subcortical infarction, the peak CMC appeared on either the healthy or the affected side of the brain. In patients with motor cortex infarction, the peak CMC emerged on the healthy side of the brain. Moreover, it is argued that alterations in the lesion site can impact CMC in stroke patients. Post-stroke brain tissue damage leads to neural network reorganization through neuroplasticity, involving the strengthening or weakening of synaptic connections between neurons to restore normal function (Chollet et al., 1991; Cramer et al., 1997; Nelles et al., 1999). The function of the impaired region is taken on by the adjacent region on the affected side or even the unaffected region to compensate for the loss of function in the impaired area (Mathiowetz et al., 1985; Chollet et al., 1991). In cases where the cortex is not directly affected, as in subcortical infarcts, its neighboring areas can compensate for the lost functions, resulting in the CMC peak appearing on the affected side, akin to the spatial pattern observed in healthy individuals (Chollet et al., 1991). In instances of cortical infarcts, the neighboring region on the affected side is inadequate to offset the loss of function and necessitates compensation from the unaffected region. These findings indicate the importance of considering the impact of the lesion site during the analysis of CMC in stroke patients, providing a crucial foundation for motor rehabilitation research based on brain-computer interfaces.

To gain a deeper understanding of the stroke’s developmental process and implement appropriate therapeutic rehabilitation strategies, stroke can be classified into acute, subacute, and chronic phases based on the time of onset and clinical presentations (Xie et al., 2022). The prevalent belief is that patients’ motor function gradually recovers during the course of stroke progression. This is possibly due to cortical reorganization taking place in neighboring or more distant regions of the affected area after a stroke, which assumes the functional roles of the impaired region, thereby leading to alterations in the spatial pattern of brain activation (Nudo, 2007; Assaf and Pasternak, 2008; Grefkes and Fink, 2011). The alterations in the spatial activation pattern of brain regions at various stages of stroke also impact the placement of the peak CMC on the brain topography in stroke patients, serving as an indicator for measuring the intensity of brain and muscle control responses. This was evidenced in a comparative study (von Carlowitz-Ghori et al., 2014). The study revealed that during the acute phase, the peak CMC was located on the healthy side of the brain, while in the chronic phase, there was no notable distinction in CMC amplitude between the affected and healthy brain sides. This suggests that in the acute stage of stroke, the healthy side of the brain assumed the primary functional activities, and the peak CMC was situated on this side. During the chronic stage of stroke, the patient’s motor function gradually improved, and the functional activity on the affected side of the brain normalized, resulting in no noteworthy disparity in CMC amplitude between the two sides during this period. The dynamic fluctuations of CMC on both brain sides in the acute and chronic stages could potentially reflect the process of motor function restoration in stroke patients.

Typically, CMC tends to increase over time during the stroke period as motor function is restored in stroke patients (Zheng et al., 2018). For instance, Krauth et al. (2019) recorded alterations in CMC in stroke patients from the acute phase to the chronic phase. The findings revealed that CMC amplitude was lower in the acute phase and gradually surpassed levels observed in healthy individuals during the chronic phase, correlating with substantial motor function recovery. This suggests that CMC has the potential to function as a biomarker during stroke recovery. However, in contrast to the findings of Krauth et al. (2019), the study by von Carlowitz-Ghori et al. (2014) demonstrated motor function improvement in stroke patients as they transitioned from the acute to the chronic phase, with no notable alterations in CMC. The variation in study results could stem from discrepancies in the motor capacity and the intensity of voluntary EMG activation in the limbs of stroke patients during participation. Prior research has established a positive correlation between CMC amplitude and EMG activation intensity (Kilner et al., 2000; Ushiyama et al., 2011). Patients in the first group exhibited diminished motor capabilities during the experiment, evidenced by a Fugl-Meyer Assessment (FMA) score of 0 for the wrist (out of a full score of 10), and weak voluntary EMG activation during limb movement. This condition might hinder the detection or quantification of synchronization between neuronal and muscular motor unit activities. Consequently, this diminishes the amplitude of the CMC. With the progression of the stroke period, patients’ motor capacity gradually improves, accompanied by a corresponding increase in EMG activation intensity, resulting in elevated CMC amplitudes. Conversely, patients in the second group exhibited superior exercise abilities during the experiment, with an average score of 4 on the Medical Research Council (MRC) scale (on a 5-point scale). During exercise, voluntary EMG activation intensity was higher, resulting in relatively higher CMC amplitudes with minimal change over time. This observation underscores the significance of the voluntary EMG signal, a crucial prerequisite for CMC measurement. Similar to previous studies’ inclusion and exclusion criteria (Guo et al., 2020), thorough consideration of motor capacity and the presence of voluntary EMG is imperative when investigating CMC in stroke patients. During the acute phase of stroke, patients often have reduced motor capacity and may be incapable of exercising, leading to minimal voluntary EMG amplitudes that impact CMC measurement. Thus, CMC application may be more appropriate for subacute and chronic stroke patients with residual motor capacity capable of generating voluntary EMG signals.

### 3.2 Rehabilitation training

Clinically, rehabilitation training for stroke patients encompasses a systematic treatment and rehabilitation program aimed at enhancing or restoring the patient’s motor and cognitive functions (Sørensen et al., 2012). CMC measures the strength of the functional connectivity between motor cortical activity and controlled muscles (Stokkermans et al., 2023), indicating the restoration of motor function after stroke. It serves as an index for evaluating rehabilitation during stroke recovery (Geertsen et al., 2013). Repeated rehabilitation training can promote the formation of new neuronal connections and synaptic plasticity in the brain affected by stroke, thereby strengthening the neural functional connections between cortical regions and motor muscles (Lin et al., 2015; Morioka et al., 2018). As the motor function of stroke patients recovers, their CMC gradually strengthens and may even surpass the levels observed in healthy individuals (Zheng et al., 2018). A study involving a brief training session in healthy individuals revealed that CMC improved shortly after the training (Larsen et al., 2016). Comparable findings were reported by Perez et al. (2006) and Geertsen et al. (2013). They noted changes in CMC corresponding to improved motor performance. To address motor dysfunction in stroke patients, the duration of rehabilitation training has been prolonged. Zheng et al. (2018) administered regular rehabilitation training (including physiotherapy and occupational therapy) for four weeks to stroke patients. They observed significant improvements in CMC after the rehabilitation period compared to before. Similarly, Belardinelli et al. (2017) carried out four weeks of exercise-based rehabilitation training in severely paralyzed chronic stroke patients and noted an increase in CMC along with improved motor performance post-exercise rehabilitation training. Ma et al. (2014) conducted a repeated follow-up study involving four cycles of rehabilitation training in stroke patients, with each cycle lasting 10 days. The rehabilitation training led to varying degrees of improvement in motor function on the affected side, accompanied by a tendency of γ band CMC on the affected side to increase with the recovery of motor function. The study suggested that continuous rehabilitation training can stimulate damaged neural circuits, facilitate neuroplasticity, aid in the establishment of new motor patterns and functional connections, and significantly enhance the level of CMC (Hu et al., 2022).

Additionally, the type and intensity of rehabilitation training should be considered. For instance, diverse electrical stimulation methods have been extensively employed in motor rehabilitation following strokes to facilitate functional enhancement by reconnecting the impaired network. These methods encompass transcranial electrical stimulation, cerebellar and spinal cord electrical stimulation, peripheral electrical stimulation (ES), and other emerging technologies (Bao et al., 2020). Notably, ES can produce sustained and sufficient sensory input, affecting the excitability of the motor cortex. Pan et al. (2018) combined hand function training with ES treatment and found that CMC was significantly increased and hand function was improved in patients with chronic stroke, this is consistent with the findings of Lai et al. (2016). NMES (neuromuscular electrical stimulation, NMES) is also extensively employed for enhancing motor function post-stroke (Howlett et al., 2015). In comparison to solely traditional rehabilitation training, the combined rehabilitation approach integrating NMES and traditional methods is more effective in enhancing motor function and improving CMC in stroke patients (Bao et al., 2021). A study that compared the impacts of constrained rehabilitation training versus traditional training on upper limb function 3–9 months post-stroke revealed that constrained rehabilitation training led to a more substantial improvement in paralyzed upper limb function among stroke patients (Goldstein, 2007). Contrary to weaker NMES intensity, which solely improves CMC in the ascending pathway (Bao et al., 2019), stronger NMES enhances CMC in both the ascending and descending pathways (Bao et al., 2021). Higher stimulation intensity of NMES at motor levels may exert a more significant modulatory effect. Electrical stimulation generates motor potentials along the corticospinal tracts, depolarizing neuronal cell membranes and subsequently activating muscle and brain barriers. This peripheral somatosensory input induced by NMES promotes motor control processes (Lai et al., 2016). Conversely, NMES surpassing the motor threshold can potentially enhance corticospinal excitability by recruiting additional synaptic motor neurons and motor reflexes (Chipchase et al., 2011). Consequently, more rigorous rehabilitation could additionally facilitate the effective reorganization of impaired bidirectional neuromuscular control circuits and bolster CMC in the ascending and descending pathways. In a meta-analysis investigating heightened rehabilitation intensity after stroke, Kwakkel et al. (2004) concluded that augmenting rehabilitation intensity led to enhancements in motor function within the initial 6 months following stroke. Hence, upcoming studies should investigate the direct impact of the type or intensity of rehabilitation training on CMC in stroke patients. Additionally, refining rehabilitation training programs and formulating personalized motor rehabilitation plans can better foster the recovery of patients’ neurological and motor functions.

### 3.3 Experimental design

Experiments examining CMC in stroke patients commence with the identification of the specific brain region, muscle, and suitable motor task. CMC is regarded as an assessment of neural oscillations within the brain and regulated muscles (Zheng et al., 2018). Neural oscillations are initiated by the coordinated activity of neurons in the brain. When the brain issues a motor command, the command information is conveyed as neural oscillations through the spinal cord to the neuromuscular tissues of the limbs. The muscle neurons then react, controlling the upper limbs to execute movements. Simultaneously, the motor feedback information from the muscle tissues in the limbs ascends through the spinal cord as neural oscillations to the sensory-motor cortex of the brain. The sensory-motor cortex subsequently incorporates the feedback information and modifies the motor commands (Conway et al., 1995). Hence, diverse movement tasks and muscles within the experimental design constitute crucial factors impacting CMC in stroke patients.

Motor tasks are typically classified into static and dynamic force outputs. Force results from intricate interactions among components of the neuromuscular system, leading to distinct patterns of corticospinal oscillations for various movements. Several studies have discovered that the motor cortex exhibits heightened β band oscillations during the control and maintenance of static force (Baker et al., 1997; Salenius et al., 1997; Gross et al., 2000; Watanabe et al., 2021), and increased γ band oscillations when generating dynamic force (Baker et al., 1997; Chen et al., 2018b). This phenomenon arises because the muscle length remains constant during static force output (Song et al., 2018), necessitating only sufficient tension to counteract the external force. Thus, the fluctuations in force complexity are minimal, and weaker neuromuscular control effects and lower-band neural oscillations (mainly β) are adequate to drive task performance. Conversely, during dynamic force output, muscles not only alter length when generating force but also swiftly integrate the visual and somatosensory information required to generate suitable motor commands. Consequently, the force fluctuation complexity is high, and the corticospinal oscillatory patterns of the sensorimotor system must shift to higher frequencies (mainly γ) to achieve the intended movement (Omlor et al., 2007; Duan et al., 2018). However, in stroke patients, damage to their cerebral nervous system affects both the transmission of regulatory commands from the motor cortex to the muscles involved in the movement and the reception of sensory feedback from the muscles and the sensorimotor system by the motor cortex. This results not only in a reduction in the relevant oscillatory activity (Peng et al., 2023) but also an abnormality in the CMC of the corresponding bands.

In stroke patients engaging in an upper limb extension dynamic force task (Fang et al., 2009), the γ band CMC of the triceps brachii (TB), anterior deltoid (AD), and biceps brachii (BB) muscles was notably lower compared to healthy controls. In healthy individuals, upper limb extension as a dynamic force task induces γ oscillations in the motor control pathway, whereas stroke patients experience relatively weaker γ oscillations due to brain damage and an inability to establish effective information communication with their muscles. Additionally, differences in CMC between stroke patients and healthy individuals are also evident in finger extension and flexion movements. In a study on cortical muscle connectivity patterns in the upper limb post-stroke (Guo et al., 2020), the CMC of the finger extensor digitorum (ED) was notably lower in stroke patients compared to healthy controls when performing a finger extension task (20% isometric maximum voluntary contraction, 20% iMVC). Conversely, during the finger flexion task completion (20% iMVC), the CMC of the ED in stroke patients did not differ from that of healthy controls. This discrepancy in CMC based on movement tasks is observed not only between stroke patients and healthy individuals but also within the stroke patient group. Bao et al. (2018) examined inter-hemispheric differences in CMC in chronic stroke patients, where stroke patients used online visual feedback to dynamically modify wrist extension. The γ band CMC on the affected side was notably lower compared to the healthy side. The γ band CMC has been linked to dynamic behaviors (Gwin and Ferris, 2012), and as stroke patients are unable to sustain coordinated dynamic activities, a reduction in the level of γ band CMC on their affected side is plausible. In an upper limb movement task for stroke patients (Chen et al., 2018b), participants were instructed to perform two consecutive maneuvers: first, slowly raising the arm to the chest and then maintaining it motionless at the chest. The former constituted a dynamic force output, whereas the latter constituted a static force output, resulting in substantial CMC in stroke patients in the γ and β bands, respectively. Furthermore, it is noteworthy that in motion tasks categorized under static force, significant CMC progressively transitions from the low band to the high band with escalating force levels (Omlor et al., 2007). Research indicates that notable CMC is observed in the γ band during higher intensity contraction tasks (Brown et al., 1998; Mima et al., 1999b). This phenomenon could be attributed to heightened cortical excitation elicited by stronger contractions, leading to shifts in the oscillation patterns of local cortical networks from low to high frequencies.

The selection of target muscles in stroke patients is critical as brain damage can lead to irregularities in neuromuscular regulation and muscle activity response, resulting in impaired signaling of brain-muscle control and feedback pathways, ultimately leading to limb motor dysfunction (Qiao et al., 2023). Based on the principle of cortical topography, the human hand and upper arm represent a significant portion of projections in the sensorimotor region (Penfield and Boldrey, 1937), making them more susceptible to functional impairment due to brain damage in stroke patients. Statistics indicate that as many as 85% of stroke patients experience upper limb motor deficits (Cantero-Téllez et al., 2019). Moreover, the hand, the most intricate motor organ in the human body, boasts exceptional dexterity, fine motor skills, and heightened sensory and tactile capabilities, and serves a crucial role in daily life (Du et al., 2022). Consequently, researchers have emphasized the upper limb muscles of stroke patients.

Muscles can be classified into active and antagonist muscles based on their functions and interactions in movement, both of which are essential for facilitating limb motion (Hobart et al., 1975). In the course of executing a movement, the cerebral cortex intricately coordinates the contraction and relaxation of the active and antagonist muscles, ensuring their collaboration to regulate the fluidity and precision of the movement (Fu et al., 2021). Stroke causes damage to cortical brain regions, impacting the precise regulation of active and antagonist muscles, resulting in compromised muscle coordination and dyskinesia, potentially leading to abnormal CMC compared to that of healthy individuals (Fauvet et al., 2021). Guo et al. (2020) discovered that during finger stretching exercises at a higher strength level (40% iMVC), the CMC peak value of the flexor digitorum (FD) in stroke patients decreased compared to that at a lower strength level (20%iMVC), while the CMC peak value of the ED increased, exhibiting a complete reversal of healthy individuals’ responses. This phenomenon may stem from reduced FD antagonism in stroke patients. Simultaneously, the weakened ED requires greater cortical involvement to generate increased power output.

Muscles in the limb can be classified into proximal and distal muscles depending on their proximity to the trunk. Proximal muscles in the upper limb are predominantly situated in the upper arm and shoulder, offering stability and assistance to the upper limb while facilitating shoulder joint movements. Distal muscles are primarily located in the forearm and hand, enabling precise movements and finger control. In a study by Mima et al. (2001) stroke patients in the chronic phase were recruited to assess their corticomuscular coherence (CMC) during grip strength, wrist extension, and elbow flexion tasks involving the thumb opponens pollicis (OP), extensor carpus radialis (ECR), and biceps brachii (BB). The study revealed that the peak CMC for OP and ECR on the affected side was significantly lower than that on the healthy side, while the peak CMC for BB did not exhibit a significant difference between the affected and healthy sides. This aligns with the clinical observation that proximal shoulder and elbow muscles tend to regain function before distal hand muscles. One possible explanation is that, as the cortical representation of distal muscles occupies a larger area than proximal muscles (Penfield and Boldrey, 1937), the dependence of distal muscles on inputs from corticospinal pathways is increased (Turton and Lemon, 1999). During a stroke, it is more probable that the region of cortical representation of distal muscles is impacted, leading to a decreased likelihood of neuroplasticity recovery. Similarly, in stroke patients engaging in isometric finger extensions (Guo et al., 2020), the peak CMC for the finger flexors (flexor digitorum, FD) is observed in the central brain region, while the peak CMC for the biceps brachii (BB) and triceps brachii (TRI) is detected in the unaffected hemisphere of the brain. This phenomenon is associated with the brain’s neuroplasticity mechanism to enlist resources from the unaffected hemisphere and other regions following a stroke (Chechik et al., 1998). However, the displacement of the peak corticomuscular coherence (CMC) for proximal muscles exhibited a more significant impact compared to the distal muscles, indicating a greater neuroplastic response of compensatory mechanisms in proximal muscles post-stroke compared to distal muscles (Guo et al., 2020).

In conclusion, the selection of movements and muscles during experimental design can significantly influence CMC in stroke patients. Therefore, in the design of experiments to investigate CMC in stroke patients, emphasis should be placed on specific frequency bands that demonstrate significant CMC based on the nature of the movement, such as prioritizing the study of β band CMC in stroke patients utilizing static force output and concentrating on γ band CMC in stroke patients using dynamic force output and higher intensity contraction. Moreover, the sequence of motor function recovery in the proximal and distal muscles of the upper extremity differs among stroke patients. The distal muscles, primarily responsible for intricate movements, exhibit delayed recovery, whereas the proximal muscles, involved in gross movements, tend to recover earlier, facilitating the execution of related motor tasks. Thus, it is imperative to rationally select the target muscles during experimental design. While ensuring stroke patients can effectively complete the experimental tasks, additional attention should be paid to the distal muscles, known for their heightened sensitivity (Rothwell et al., 1991; Fu et al., 2021), to effectively identify the aberrant motor function mechanisms in stroke patients.

## 4 Challenges and future prospects

Recent advancements in the field of neuroimaging techniques and neurocomputational modeling have significantly advanced the research on CMC in stroke patients. These techniques enable researchers to gain a deeper understanding of the interplay between the cerebral cortex and muscles, thereby offering valuable insights for enhancing the recovery of neurological function and the rehabilitation of motor function in stroke patients. However, several inquiries still require further exploration.

Initially, there continues to be an ongoing debate on the necessity of applying EMG rectification in CMC studies. Initial studies suggested that preprocessing of implementing EMG rectification might improve the temporal precision of action potentials, thereby amplifying the significance of β band CMC (Reyes et al., 2017; Chen et al., 2020). Myers et al. (2003) were the first to propose this notion, which was based on a simulation study devoid of empirical data. Additionally, Farina et al. proposed that EMG rectification might enhance the detection of β band CMC (Farina et al., 2013). Nevertheless, Yao et al. (2007) examined the impact of EMG rectification on power and coherence spectra utilizing EEG and MEG signals. Their findings indicated that EMG rectification potentially enhanced the recognition of motor unit discharge rates. Nonetheless, the outcomes of cortical muscle coherence estimation using both corrected and uncorrected EMG signals did not exhibit significant differences. Similar conclusions were drawn by Bayraktaroglu et al. (2011) and Yang et al. (2016a), among others. Secondly, the majority of ongoing research investigating the impact of force level on CMC in stroke patients has employed maximum voluntary contraction (MVC) as the standard measure for force levels. However, it is crucial to consider that the level of force output varies among stroke patients. Particularly during the acute phase of stroke onset, patients have severely restricted exercise capacity, and a certain proportion of MVC may result in a notably low force output level (von Carlowitz-Ghori et al., 2014), thereby influencing the anticipated experimental outcomes. Furthermore, while the motor system of stroke patients is impacted, the majority do not exhibit any tactile deficits. Employing MVC as the benchmark for the target force level, subjects may have restored some motor capacity during subsequent assessments, potentially leading to heightened tactile stimulation. The potential inaccuracy of experimental results arises from the unknown impacts of the feedback afferent pathway on CMC (Riddle and Baker, 2005; Baker, 2007).

In general, numerous aspects of CMC research in stroke patients warrant further exploration and development. Firstly, a dearth of studies exists concerning the influence of various stroke types on CMC analysis methods. Ischemic stroke involves vascular occlusion resulting in ischemic injury, potentially leading to neurological damage and functional alterations. In hemorrhagic stroke, the rupture of a cerebral blood vessel with hemorrhage can cause more pronounced functional changes in the nervous system, posing greater challenges to the applicability of CMC analysis methods. Subsequent studies should conduct more comprehensive research on the distinctive attributes and clinical presentations of different stroke types, evaluating their effects on CMC analysis methods and aiming to enhance the precision and applicability of such analytical approaches. Secondly, despite the prevalence of lower limb motor dysfunction in nearly 80% of stroke survivors, the majority of current CMC studies in stroke patients have concentrated on upper limb assessments, neglecting comprehensive investigations into the functional connectivity between the brain and lower limbs. The initial investigation into lower limb CMC post-stroke was conducted by Xu et al. (2023), revealing a considerable reduction in lower limb CMC among stroke patients compared to healthy controls. These findings mirror outcomes from numerous studies focusing on the paralyzed upper extremities of stroke patients. Furthermore, examining CMC disparities between proximal and distal muscles in the lower limbs of stroke patients may shed light on whether comparable phenomena and mechanisms are present in the lower limbs as observed in the upper limbs. Lastly, the influence of visual feedback on CMC warrants examination. The vision serves as the precursor and guide for all our motor plans (Flanagan and Johansson, 2003). Due to their inherent motor dysfunction, a majority of stroke patients tend to depend heavily on visual feedback during exercises to enhance the precision of motor execution. Research has demonstrated an augmentation in β band CMC during motor tasks with visual guidance (Nijhuis et al., 2021). Furthermore, the impact of age on CMC is contingent upon the visual feedback accessible to the participant (Watanabe et al., 2020). Consequently, anticipations for further advancements in CMC research are high, promising novel pathways for addressing neurological disorders and facilitating deeper understandings of human motor control and the acquisition of novel movements.

## Author contributions

ZG: Writing – original draft, Writing – review & editing. SL: Writing – original draft. XR: Writing – original draft. YuW: Writing – original draft. MX: Writing – original draft. JW: Writing – original draft. MQ: Writing – original draft. YiW: Writing – original draft. ZS: Writing – original draft. ZZ: Writing – review & editing. YZ: Writing – review & editing. XZ: Writing – review & editing. YY: Writing – review & editing.
